# Solving the Problem of Building Models of Crosslinked Polymers: An Example Focussing on Validation of the Properties of Crosslinked Epoxy Resins

**DOI:** 10.1371/journal.pone.0042928

**Published:** 2012-08-20

**Authors:** Stephen A. Hall, Brendan J Howlin, Ian Hamerton, Alex Baidak, Claude Billaud, Steven Ward

**Affiliations:** 1 Chemical Sciences Division, Faculty of Health and Medical Sciences, University of Surrey, Guildford, United Kingdom; 2 Cytec Engineered Materials, R422 The Wilton Centre, Cleveland, Ohio, United States of America; King’s College London, United Kingdom

## Abstract

The construction of molecular models of crosslinked polymers is an area of some difficulty and considerable interest. We report here a new method of constructing these models and validate the method by modelling three epoxy systems based on the epoxy monomers bisphenol F diglycidyl ether (BFDGE) and triglycidyl-*p*-amino phenol (TGAP) with the curing agent diamino diphenyl sulphone (DDS). The main emphasis of the work concerns the improvement of the techniques for the molecular simulation of these epoxies and specific attention is paid towards model construction techniques, including automated model building and prediction of glass transition temperatures (T_g_). Typical models comprise some 4200–4600 atoms (*ca.* 120–130 monomers). In a parallel empirical study, these systems have been cast, cured and analysed by dynamic mechanical thermal analysis (DMTA) to measure T_g_. Results for the three epoxy systems yield good agreement with experimental T_g_ ranges of 200–220°C, 270–285°C and 285–290°C with corresponding simulated ranges of 210–230°C, 250–300°C, and 250–300°C respectively.

## Introduction

Epoxy resins are one of the more commonly encountered families of commercial thermosetting polymers and are widely used in a diverse range of industrial applications including coatings, adhesives, electronic devices, and as the matrix resin for advanced structural composites (*e.g.* aerospace automobiles, marine vessels and space vehicles) [1], [2], [3]. Along with other thermosetting polymers, epoxies generally form highly-branched, three dimensional network structures. The complexity of such a structure, with its inherent heterogeneity and poor solubility, presents many challenges to the analyst attempting to obtain chemical characterisation data. Molecular modelling is a powerful tool for understanding structure derived chemical and physical properties [4]. It has been used with success in the past to derive a range of physical and mechanical properties of polymers, including Young’s Modulus of Elasticity, Poisson’s Ratio and Lamé constants as well as the glass transition temperature. The method for modelling polymer systems used by Gu *et al.* [5] Fan *et al,* [6], Ford *et al*. [7] and Gou *et al*. [8] creates an oligomer by chain growth. The way the system is created results in a more natural model with greater amorphous characteristics, but still possessing a degree of molecular strain. Oligomer and (if necessary) cross linking molecules are packed into a periodic cell to a specified density manually or by an algorithm such as Amorphous Cell from Materials Studio. Once this is done, final cross linking can be completed through residual, un-reacted functional groups, followed by energy minimisation and molecular dynamics (MD) to relax the structure.

Molecular modelling of polymers is a growing area and it has been used in a wide variety of polymeric systems. By far the most effort has been concentrated on epoxies, owing to their general usefulness. Reports have predicted the structure, mechanical properties and moisture diffusion in epoxy resins [6], [7], [9]–[11]. Other thermosetting polymers have also been modelled including, polycyanurates [12], polybenzoxazines [13], polyimides (in particular gas permeation across polyimide membranes) [14]–[16] and cyclohexanone formaldehyde resins (plastic printing) [17]. Non thermosetting polymers have included polyethylene oxides [18], polysiloxanes (glass transition temperature) [19], [20] and polyethylene terepthalate (gas diffusion) [21]. Recently the field has moved into the modelling of nanocomposites with carbon nanotube reinforced composites becoming of interest [22], [23].

It is fundamental to our approach that the simulations that are performed are always supported by empirical data, either single crystal data in the formation of structures or from physical or mechanical measurements when determining properties for the final polymer. In this paper we report the latest development of our modelling techniques both in regard to a new method for the automatic generation of cross-linked atomistic three dimensional molecular models and the determination of T_g_ using non-subjective methods. Furthermore this paper covers crosslinked polymers and the application of Materials Studio [24] to the determination of the physical and mechanical properties of three crosslinked epoxy resins. This method is generally applicable to all crosslinked resin systems and in future publications we will demonstrate this method with cyanurate and benzoxazine polymers, which are currently of great interest in microelectronics.

## Methods

### Materials

**Figure 1:**
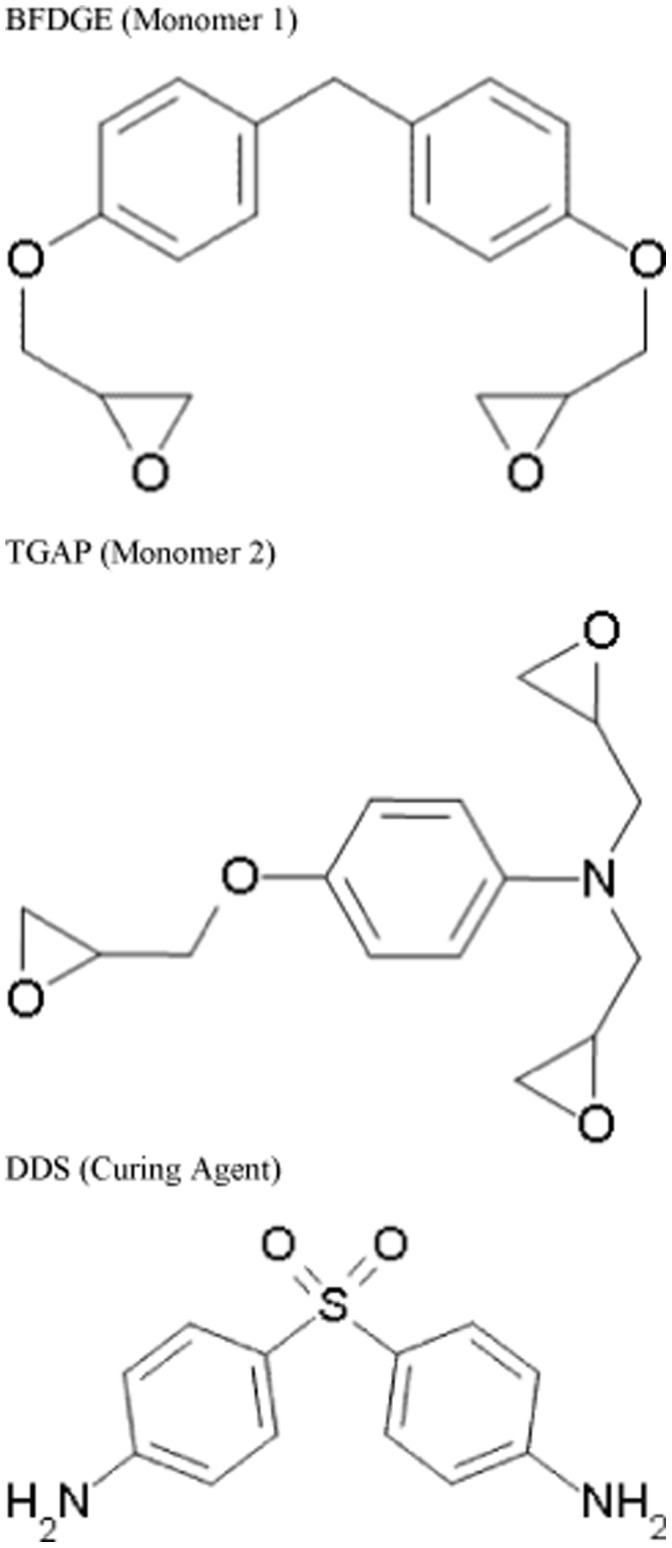
Structures of chemicals used in this work.

All the epoxy resins used in this work, as part of physical preparations or atomistic simulations were made from the diamine, DDS, and the epoxy monomers BFDGE and TGAP (Figure 1 and Table 1).

**Table 1:** Chemicals used in this work.

Chemical	EEW*	Purity	Impurities	Isomers
BFDGE	162	96%	Appear to be monoepoxide and oligomers	38.6% p,p'47.0% o,p'10.6% o,o'
TGAP	96	96%	Appear to be functionalised oligomers	–
DDS	n/a	97%	–	–

* Epoxide equivalent weight.

### Sample Preparation and Thermo-mechanical Analysis

**Figure 2:**
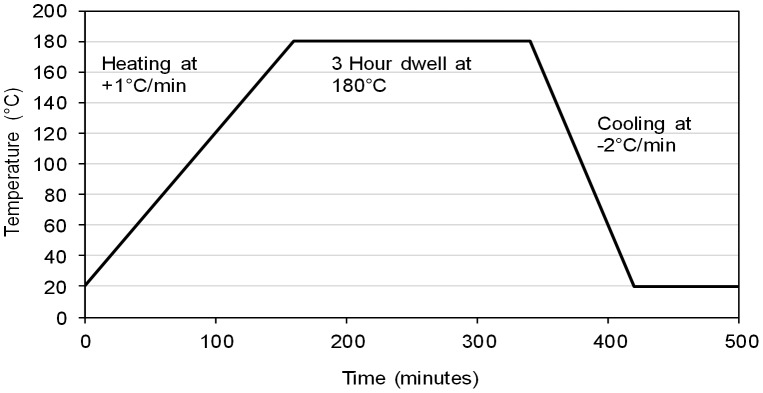
Schematic showing cure schedule used for all epoxy formulations.

The three epoxy formulations (Table 2) were mixed to yield individual samples (*ca.* 100 g) and >90 g was cured following the scheme in Figure. 2. The remaining material was kept as uncured mix. Samples (*ca.* 5 mg) were taken for Differential Scanning Calorimetry (DSC) from the cured and uncured material. A cured and an uncured sample was taken from each formulation and scanned from −50°C to 350°C at a rate of 10 K/minute under nitrogen (40 cm^3^/min.). DMTA was carried out on cured neat resin samples (4×1.4×40 mm^3^) in air using an ARES LS 2K/2K FRT DMTA in torsion rectangular solicitation mode at 3 K/minute (0.1 Hz frequency and 0.1% strain). Specimens were dried prior analysis (100°C over night if T_g_ >180°C).

**Table 2:** Epoxy formulations used in simulations and castings in this work.

Physical Casting (1)	Physical Casting (2)	Physical Casting (3)
71.5 g – BFDGE28.5 g – DDS	60.6 g – TGAP39.4 g – DDS	67.2 g – TGAP32.8 g – DDS
Cast into 3 mm thick plate	Cast into 3 mm thick plate	Cast into 3 mm thick plate

### Molecular Modelling Software

The Materials Studio molecular modelling suite (Accelrys Inc.) was utilised in this work [24] using in house PCs (*e.g.* a Dell PowerEdge 1950, 2×Quad Core Intel Xeon E5140 2.33 GHz, 8GB RAM, 500 GB HDD). The Discover module was used for general simulation requirements, such as geometry optimisation and molecular dynamics as well as molecular mechanical analysis to predict values for tensile, bulk modulus, shear modulus, Poisson’s ratio and the Lamé constants. The Amorphous Cell module was used to build amorphous, homogenous 3D cells composed of molecules that were drawn *in silico*. It also has a number of protocols designed to make greater use of the Discover module, of specific interest is the temperature cycling protocol, which can be used for T_g_ prediction. All simulations were performed in the bulk state, i.e. without the addition of solvent as there is no added solvent in epoxy resin cure.

### Model Preparation

Formulations were made with BFDGE and Daminodiphenylsulphone (DDS) (Epoxy 1) and with TGAP and DDS (Epoxies 2 and 3). A summary of the epoxy formulations and atomistic models is given in Table 3.

**Table 3:** Epoxy systems used in this work.

Epoxy 1	Epoxy 2	Epoxy 3
30x BFDGE p,p' Monomer38x BFDGE o,p' Monomer8x BFDGE o,o' Monomer1x BFDGE o,p' – p,p' Dimer1x BFDGE o,o' – p,p' Dimer39x DDS	72x TGAP Monomer2x TGAP Trimer56x DDS	72x TGAP Monomer2x TGAP Trimer42x DDS
(119 monomers 4555 atoms)StoichiometricCured to 69% with automatic cure program.	(132 monomers 4634 atoms)StoichiometricCured to 71% with automatic cure program.	(118 monomers 4228 atoms)Epoxy excessCured to 70% with automatic cure program.
BFDGE purity: 95.5%BFDGE EEW: 162	TGAP purity: 93.3%TGAP EEW: 96.0	TGAP purity: 93.3%TGAP EEW: 96.0

### Molecular Model pre-treatment

Each model was subjected to a Discover Molecular dynamics single run using the NPT Ensemble for a minimum of 500 ps, a timestep of 1 fs, using the Anderson Thermostat at a Pressure of 0.1 MPa under the Parrinello Barostat. The force field was the Polymer Consistent Force Field (PCFF) with Atomic vdW and Coulomb Summation with a cutoff of 10 Å, a spline width of 3 Å and a buffer width of 1 Å. The same procedure was then applied to the MD simulation over a predetermined temperature range, with decrements of 10 K from the starting temperature (which was typically *ca.* 150 K above the experimental T_g_ as determined by DMTA). Each temperature step was of 125 ps with the first 25 ps of data discarded.

## Results and Discussion

### Discussion of Epoxy Equivalent Weight

**Figure 3:**
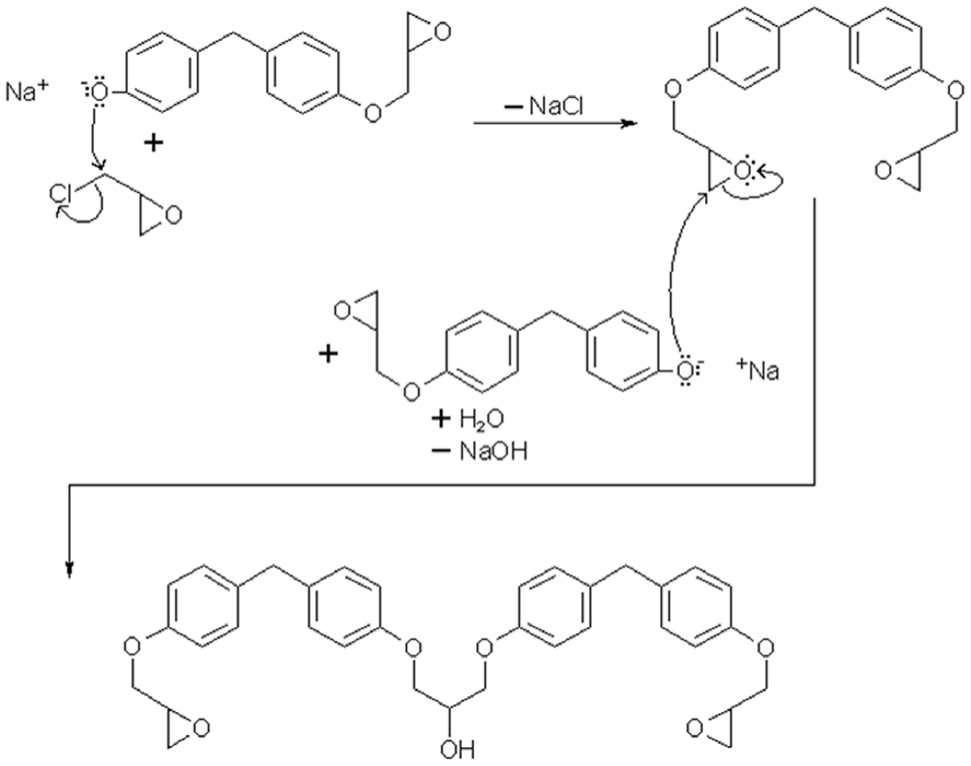
Reaction mechanism showing formation of BFDGE monomer and dimer.

With reference to Figure 3 it can be seen how epoxy monomers can be produced, with Bisphenol diglycidyl ether (BFDGE) as the example. It can also be seen how a small proportion of oligomers can be formed from the reactive species in manufacture. The phenol group is reduced by a suitable base, this base would preferably react with an epichlorhydrin molecule to form BFDGE. However under some circumstances, this reactive species will react with a BFDGE molecule to form a dimer [25]. The monomers used to prepare the physical epoxy resin samples were supplied as 96% pure, so it would not be accurate to build the atomic simulations from 100% pure monomer. From the specification of BFDGE *“Epoxy Equivalent weight (EEW) = 162 g/mol and a purity degree of 96%. The impurities appear to be monofunctionalised and oligomers.”* and from the specification of Triglycidylaminophenol (TGAP) *“EEW = 96 g/mol and a purity degree of 96%. The impurities appear to be functionalised oligomers”.* The simplest way to achieve the specified EEW is to include a proportion of functionalised dimer or trimer using equation (**1**), however this may require a greater proportion than the 4% allowed for impurities. From experience and intuition, keeping EEW close to the specification would create a simulated mix with more accurate properties than if purity was maintained with disregard to EEW. Obviously the ideal system would have an accurate EEW and purity, but to do this requires the 4% of impurity to have a precise EEW while only using whole numbers of molecules. Often keeping whole numbers of molecules will require multiplying up the molar fractions, leading to oversized models, which may be beyond the processing power available.
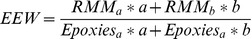
(1)


### Determination of the Model Composition Based on EEW

It would be simple to build the atomistic models using the pure monomer molecules in Table 1, but more data were available on the nature of the monomers, and this was taken into consideration when designing the models. The aim was to achieve maximum accuracy of the network within the capacity of the processing power available. The specifications for these three chemicals are summarised in Table 1, any unknown details, such as isomer fractions were taken as ideal (as per pure monomer molecule in Table 1). It is desired to achieve the empirically measured effective EEW for both BFDGE and TGAP (162 and 96 respectively), which are higher than the pure monomers (144 and 92 respectively). Units are [atomic mass units] per [epoxy functional group]. Without making the model too complex, it is possible to increase the EEW by introducing a fraction of low MW oligomer into the mix. The proportion is calculated by solving the simultaneous equations (**2**) and (**3**).
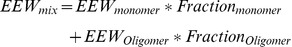
(2)

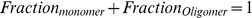
(3)


For the best accuracy the Fraction_monomer_ should be equal to the purity of the monomer, it is a molar fraction, not to be confused with the mass fraction, which is usually quoted for purity. However this can be limiting as it requires a precise EEW_oligomer_ to achieve the desired EEW_mix_. Calculation shows that 96% BFDGE monomer and 4% BFDGE dimer gives an EEW_mix_ of 161.3 (empirical value = 162) and 96% TGAP monomer and 4% TGAP octamer gives and EEW_mix_ of 96.1 (empirical value = 96). It would be possible to represent exact purity and EEW by also including a small fraction of trimer to BFDGE and a small fraction of pentamer to the TGAP mix. This solution often requires fine tuning of fractions and requires a very large model to keep the ratios of molecules as integers. For practical purposes, the model size has to be kept in check, to do this, only BFDGE dimers and TGAP trimers were used to represent the impurity molecules. It was considered more important to maintain the EEW and it can be seen in Table 3 that the final molecule ratios give the correct EEW at slight cost to purity accuracy.

### Manual Model Building and Curing

Once a model has been planned and the types and quantities of each molecule, including monomers, isomers, oligomers and monofunctionalised monomers have been calculated, one of each type of molecule needs to be drawn out in the 3D modelling software manually. The Amorphous Cell [26] module in Materials Studio can then be used with specified quantities of each of these molecules to create a 3D periodic cell of desired density and temperature. During the earlier stages of this study, this amorphous cell of monomers was then developed manually to react suitable epoxy and amine groups together while occasionally running energy minimisation and molecular dynamics to keep the model stable and to allow functional groups to diffuse together. As the available computer power has increased, it has been possible to work with larger models, which is desirable as these larger models have been shown to equilibrate better and give more accurate results [12], [13]. It should be noted however, that MD simulations can be very computationally intensive, and accurate results can sometime take weeks or months to produce [13]. It is not the only method available, others, wherein the researcher is more closely involved with the mathematics of the model include group interaction modelling [27] and atomic additivity [28]. The down side to working with larger models is that they are more difficult to create within a graphical interface. Thus, it has become necessary to create an operation that can automatically create a cured system based purely on input parameters, without the researcher having to actually deal with the choosing, making and breaking of bonds. Although models of over 6000 atoms have been made before [13], they were built by multiplying copies of a smaller cell of around 250 atoms. When it was important to build a single amorphous unit cell of *e.g.* 4555 atoms, with 119 epoxy-amine pairs requiring bonding for a 70% cure, the desire for an automatic cure program became a requirement.

### Automatic Model Building Software

**Figure 4:**
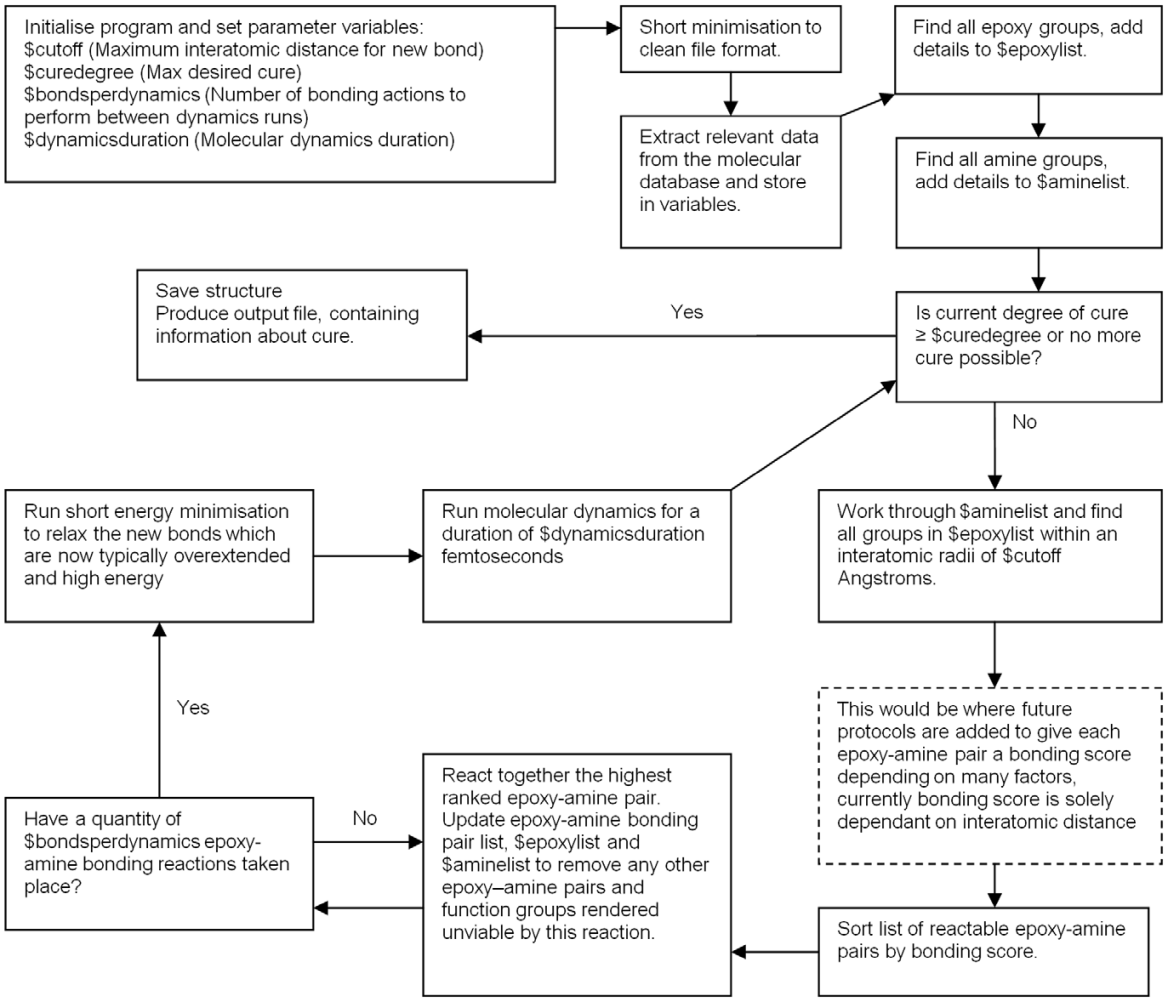
Schematic showing flow diagram for operation of automatic cure program.

Materials Studio includes the scripting language BTcL which allows deeper interaction and automation of the Discover and Amorphous Cell modules. BTcl is an extension of the open source tool command language (Tcl) developed by John Ousterhout in 1988 and carries all the Tcl operations with integration of the Discover control commands (Figure. 4). This was of great interest for automating the model construction phase as the repetitive decision making and bonding manipulation is quite transferable into code and can be performed significantly faster than it could be done manually [29].

The periodic cell packed with uncured monomer created by Amorphous cell is taken as the starting point for the program. The user definable variables are set up to determine how the model will cure and the program is set to run its course to produce an atomistic model of cured epoxy resin, ready for further investigation. The additional advantages on top of the reduction in time to produce a model also become apparent, a program will build more amorphous nature into the unit cell, as human randomness in choosing which groups to bond is a weigh off between poor quality and excessive time consumption. Also, when curing manually, it is necessary to bond a reasonable number of groups at once before running a molecular dynamics simulation to save having to come back to the model too frequently. With a program however, if it was desired, this same molecular dynamics time could be divided up so the model can relax and move after every individual bond creation.

In the current version, when the program looks through the model to find which epoxy – amine pairs to bond together, only the interatomic distance is considered, with the closest pairs taking priority and being bonded first. However it has been written so that other influences can be programmed in at a later date. For example, a more reactive primary amine could be offered higher bonding priority over secondary amines. Factors influencing bonding which would have been very difficult to include if the model was cured manually are simple to implement, for example atomic velocity or local energy could be included in the algorithm, perhaps giving priority to groups with greater mobility.

**Figure 5:**
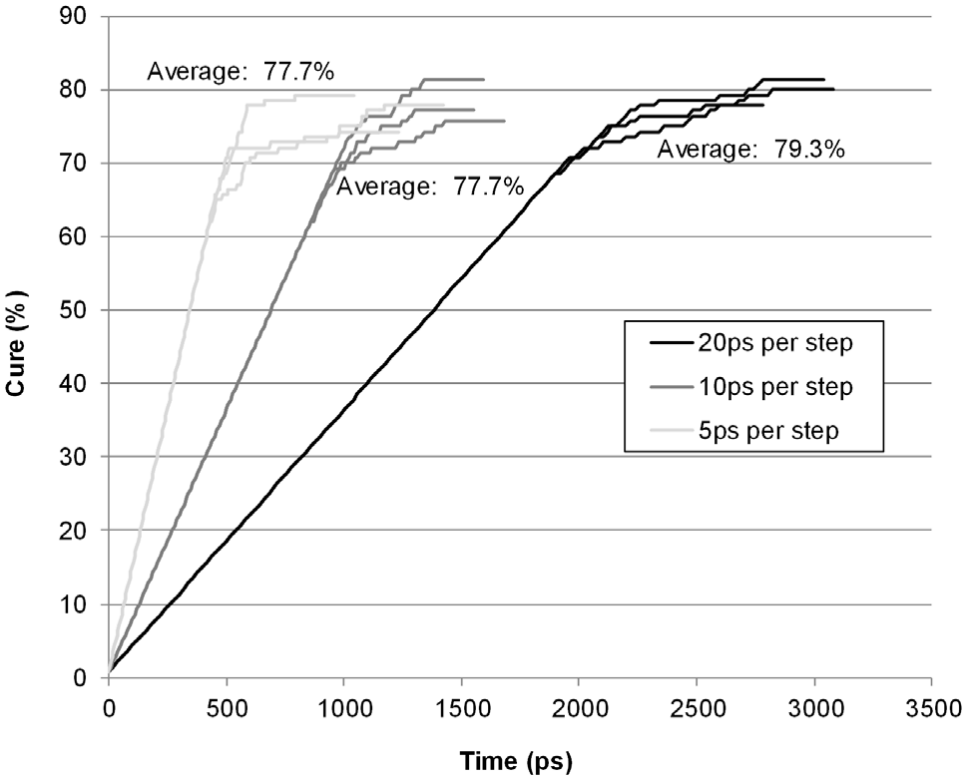
Comparison of different cure times and the degree of cure achieved.

During the automatic cure programme a 5 ps MD simulation was run after every new group was bonded. Observing the MD playback animation, it can be seen 5 ps is well in excess of the required time for the new bond and the vibrations in the surrounding structure to stabilise. When 70% of the amine groups had reacted the cure was halted after the final energy minimisation and 5 ps MD. A plot of the effect of varying the MD timestep on the degree of cure is given as Figure 5. It can be seen that increasing the time step increases the degree of cure to approximately 80% but interestingly the degree of cure never rises above 80%. Commercial epoxy cure is subject to B staging where the material is held for long period of time at an elevated temperature to achieve higher cross link density. The simulations are showing the reason for this as when diffusion control operates it is difficult for reactive groups to encounter each other to increase the cross link density.

**Figure 6:**
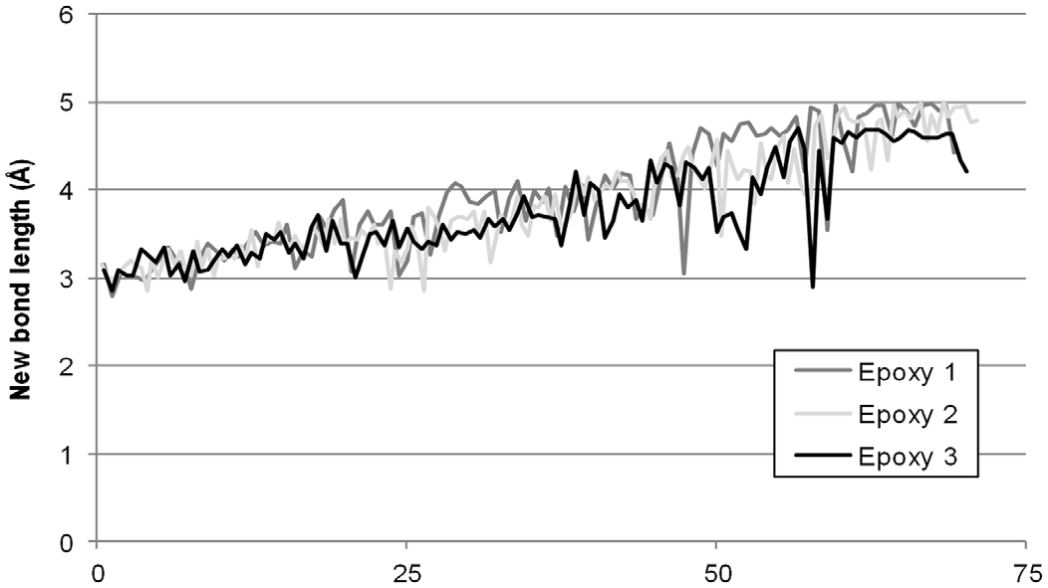
Interatomic distance between nitrogen and carbon that will be used for the new bond in automatic cure.

The interatomic separation between the nitrogen and carbon atoms of the new bond was recorded before energy minimisation. These distances were plotted alongside the degree of cure for the 3 models in Figure. 6: once the new bond has relaxed it should be close to 1.47 Å between atomic centres.

**Figure 7:**
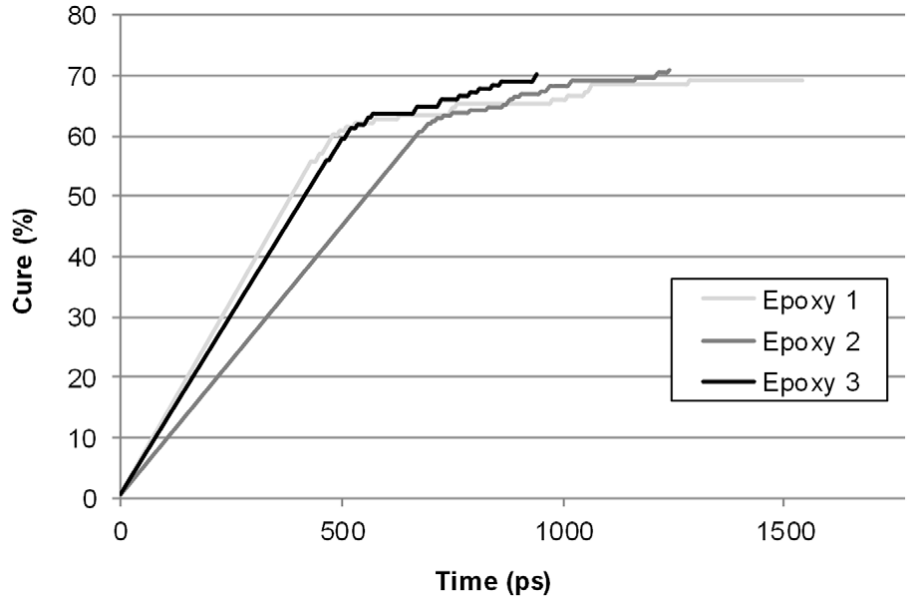
Plot of polymer conversion *vs.* elapsed dynamics time for the three epoxy models.

It is valuable to compare the interatomic bonding distances between the two TGAP based epoxies, Epoxy 2 and Epoxy 3. Epoxy 3 has an excess of epoxy groups compared to 2, so there is more choice for the amine groups, and so a greater possibility of finding bonding groups in closer proximity to any specific amine group. However care is needed when comparing stoichiometric with non-stoichiometric cures, as both models could have 70% of the amine cured, but they are not equivalent. All three models show the clear trend, as the degree of conversion increases it becomes less and less likely for an amine and epoxy to be in close proximity, with bonding distances increasing. As the cure progresses, molecular dynamics allows oligomers and chains to move, but the more bonding actions that take place, the less freedom amine and epoxy function groups have. Eventually the groups are too far away to bond and the epoxy model has cured to a degree where they do not have the freedom to diffuse closer together. When there are no functional groups within 5 Å (4.7 Å for Epoxy 3) that are capable of undergoing reaction, the model is subjected to further molecular dynamics to give the chains more time to diffuse. Figure 7 more vividly represents the difficulty the models suffered finding local functional groups with which to react towards the end of cure.

Work is currently underway to investigate whether it is possible to predict a maximum degree of cure for a given cure temperature by observing the point at which gelation happens, and the ensuing restriction in chain motion.

### T_g_ Simulation by Molecular Dynamics

**Figure 8:**
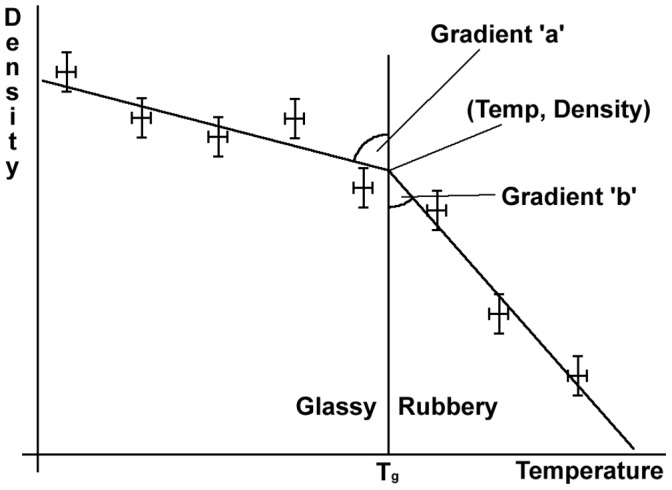
Example plot of Density *vs.* temperature, showing T_g_ and how the hinged line is fitted to data points.

At sufficiently low temperatures, polymers are in the glassy state, here the polymer chains are quite restricted in movement, with only small vibrations occurring. As the polymer is heated, there comes a point where there is sufficient energy for larger motions involving the polymer backbone, involving an estimated 20–50 chain atoms to take place [30]. Upon reaching this transition temperature, and as a result of the increased mobility, there are a number of measurable changes in physical properties. Most noticeable is that the polymer is no longer glassy, but is now in the rubbery state which will have markedly reduced stiffness, a property which can be measured using dynamic force thermo-mechanical analysis [31]. This increased mobility of the chains will also require a larger free volume between the atoms, and so the density will need to decrease to compensate. If the density is plotted against temperature for a modelled polymer system a graph similar to that in Figure 8 will be obtained. From this the glass transition temperature can be measured as the point of gradient change.

Molecular dynamics can be utilized with a molecular model to estimate T_g_ with little knowledge of the polymer chemistry involved with the glass transition. Molecular dynamics will simulate the location and velocity vector for each atom within the model over time at specified conditions. This method can be used to calculate T_g_ by running simulations at various temperatures and taking readings for density. These data can then be used to plot a graph, T_g_ can be estimated as the point of intersection between the thermal expansion gradients above and below the glass transition temperature [32].

### Investigation into Selection of MD Simulation Experiment Parameters

Berendsen and velocity scale thermostats were deemed not suitable for property determination molecular dynamics because they are too crude [33]. However, in contrast the Parrinello barostat is ideally suited for T_g_ determination because it allows for anisotropic deformation of the cell. This is acceptable because the cross linked epoxy is structurally sound. Parrinello allows more degrees of freedom, and a more natural simulation. The default thermostat and barostat values appear to be reasonably well tuned for our system, although with this system the Nosé thermostat failed to maintain a suitably stable temperature. This was not entirely surprising because the Nosé thermostat is known to have trouble maintaining equilibrium with stiff systems; a Nosé Hoover Chain would perhaps have offered some potential, but it was unavailable [34]. Experiments were performed using velocity scale to quickly reach equilibrium and showed that the unnatural scaling used by velocity scale distorted the model, resulting in poor stability in the following experiment, as the system tried to return to natural dynamics. The Anderson thermostat was chosen as it performed best in experimental trials.

Increasing the simulation resolution from 1 fs to 0.5 fs generally improved stability, although the cost of processing time for the small improvement over 1 fs made this undesirable, as the same processing time could be spent extending the duration to more than double, which would improve the average, but not the standard deviation. Increasing the cell size dramatically improved standard deviation for variance in temperature and density, but if the cell units are multiplied by 8, the processing time will take longer than 8 times that of a single cell to cope with the demands of the larger cell.

As an early improvement over previous techniques used in our group [12], [13], an automated system was used to run the molecular dynamics simulations at a number of different temperatures using the capabilities built into the Amorphous Cell module (Table 4).

**Table 4:** Simulation temperature ranges for the three epoxies.

Polymer	Temperature range
Epoxy 1	75–275°C
Epoxy 2	227–347°C
Epoxy 3	232–353°C

Data were taken from each temperature step for average and standard deviation of Temperature and Density, which were plotted graphically and analysed by an in-house program to determine the point of gradient change, which occurs at T_g_.

### Data Analysis for Obtaining Simulated T_g_

As the best fit line is not simple, it is a ‘hinged line’, it was considered worthwhile to see whether the data interpretation could be improved and automated (*i.e.* a program which could take a group of temperature and density data points and return the best fit, and a value quantifying it). This is quite complex, but returns the best possible results.

**Figure 9:**
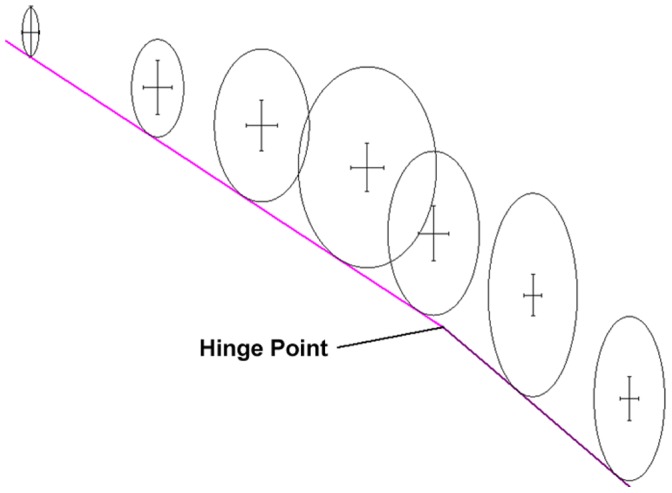
Example showing how ellipse aspect ratios are linked to error bars and radii are linked to geometry of best fit line.

Consider Figure 9 to aid understanding of how the best fit line is manipulated. For each set of density and temperature points, a range of fittings is attempted with the hinge point every 1 or 2 K along the Temperature axis. Each point is considered as a hinge, and along with the lines to be fitted can be translated up and down the density axis, gradients ‘a’ and ‘b’ can also be varied. So the line is only restricted to one extent, the hinge point must be at a fixed temperature. Once the best fit for a specific temperature is found, the process is repeated at different temperatures to allow a graph to be plotted of fit quality *vs.* temperature. The T_g_ can be read off this graph when the fit quality is at a maximum.

It was desired to achieve the best possible fit for the hinged line to the data points, with each data point accurately influencing the shape of the hinged line. With reference to Figure 9, at each data point, an ellipse was centred, which was of the same eccentricity as the standard deviation error bars and of sufficient radius to make a tangent with the best fit ‘hinged line’. The line was fitted using a Box-Behnken refinement method [35] to minimise the total area of the ellipses. To quantify the quality of fit, a straight, unhinged line is also fitted through the data and the total ellipse area calculated for this line. By dividing the total ellipse areas for the hinged line by those of the straight line, a ‘goodness of fit’ coefficient between 0 and 1 is found, which is not unlike the R^2^ vlaue for a straight line. Once calculated for a number of temperatures, these coefficients can be overlaid on the original density *vs.* temperature data. In these diagrams the red line represents the quality of the fit and the data points are for individual or group dynamics runs with standard deviation error bars in black. This fitting process therefore takes into account the uncertainty in both the simulated density and temperature and finds the turning point of the data. This is represented as the red line on the plot and therefore finds the calculated T_g_ value by taking into account the error in the data automatically.

**Figure 10:**
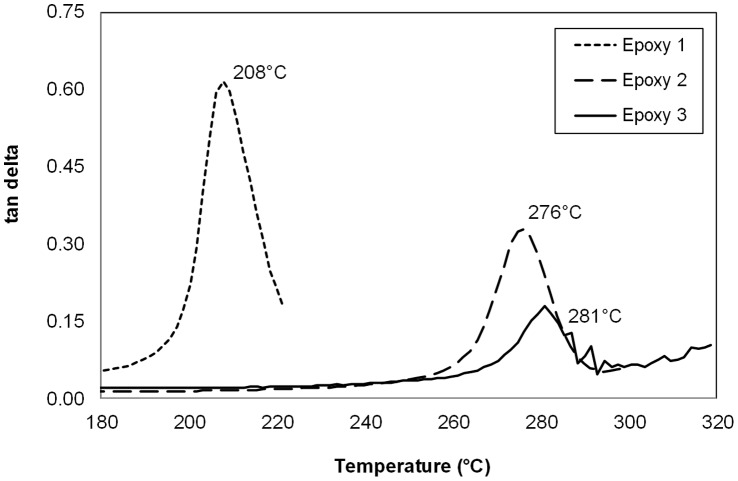
Experimental DMTA data for the three cured epoxy resins.

### Dynamic Mechanical Thermal Analysis (DMTA) of Cured Epoxy Resins

The thermomechanical data for the three cured epoxy systems (Epoxy 1, 2, and 3) are presented in Figure 10, from which it can be seen that as anticipated Epoxy 1 displays a markedly lower T_g_ (208°C as determined by tan δ_max_), due to the lower crosslink density generated by the difunctional epoxy monomers, whereas Epoxies 2 and 3 containing significant quantities of the trifunctional TGAP, show similar peak maxima (T_g_), albeit with quite different damping behaviour (as evidenced by the shape and half peak height of the tan δ peak).

### T_g_ Simulation Results and Analysis

**Figure 11:**
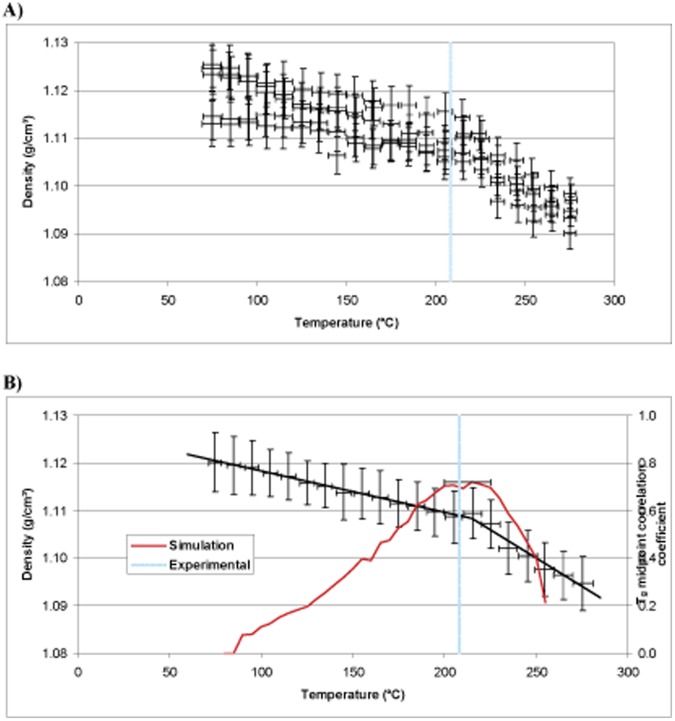
MD simulation for Epoxy 1 showing (A) raw data and (B) group data, experimental Tg superimposed in blue and prediction in red.

**Figure 12:**
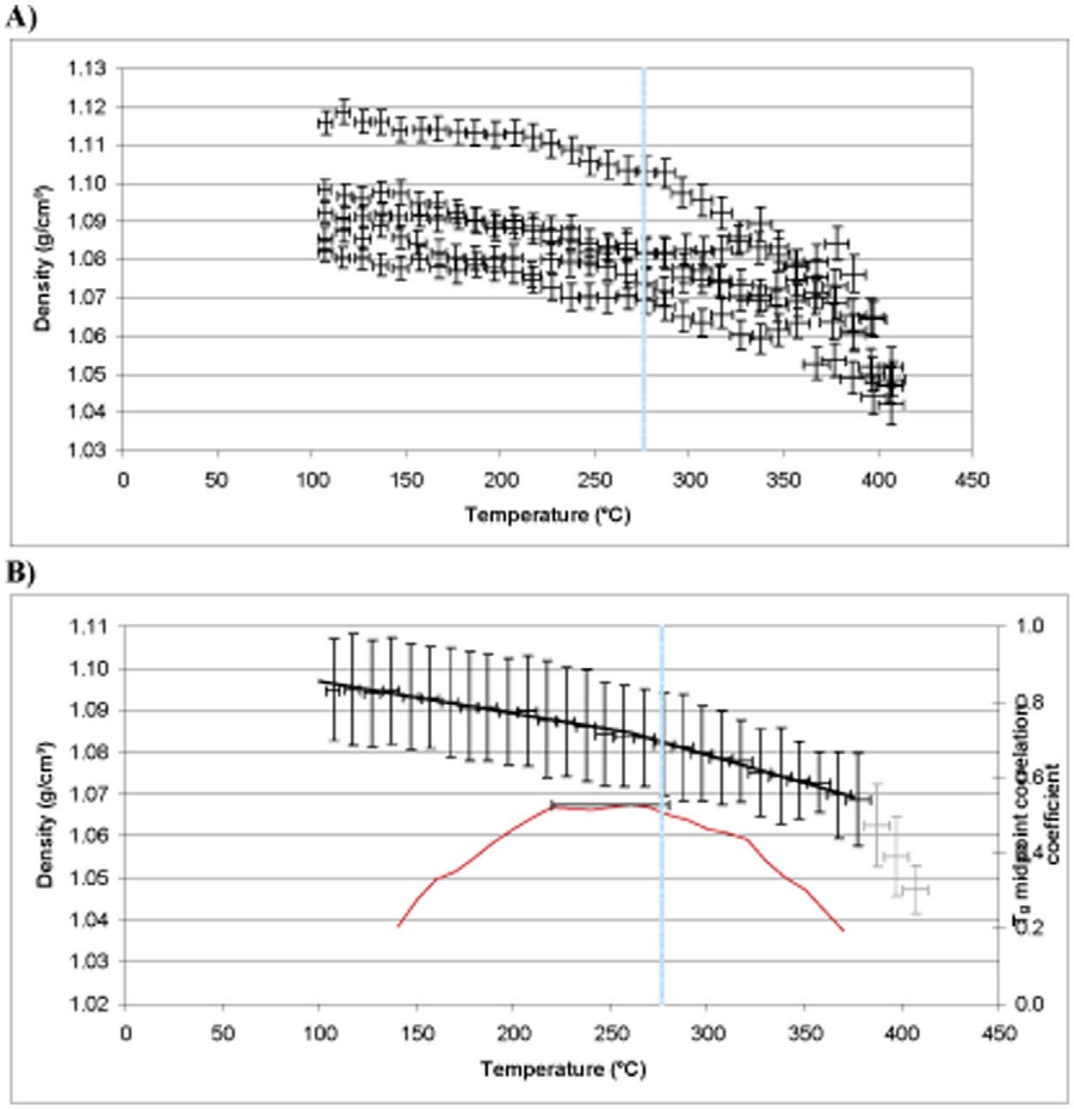
MD simulation for Epoxy 2 showing (A) raw data and (B) group data, experimental Tg superimposed in blue and prediction in red.

**Figure 13:**
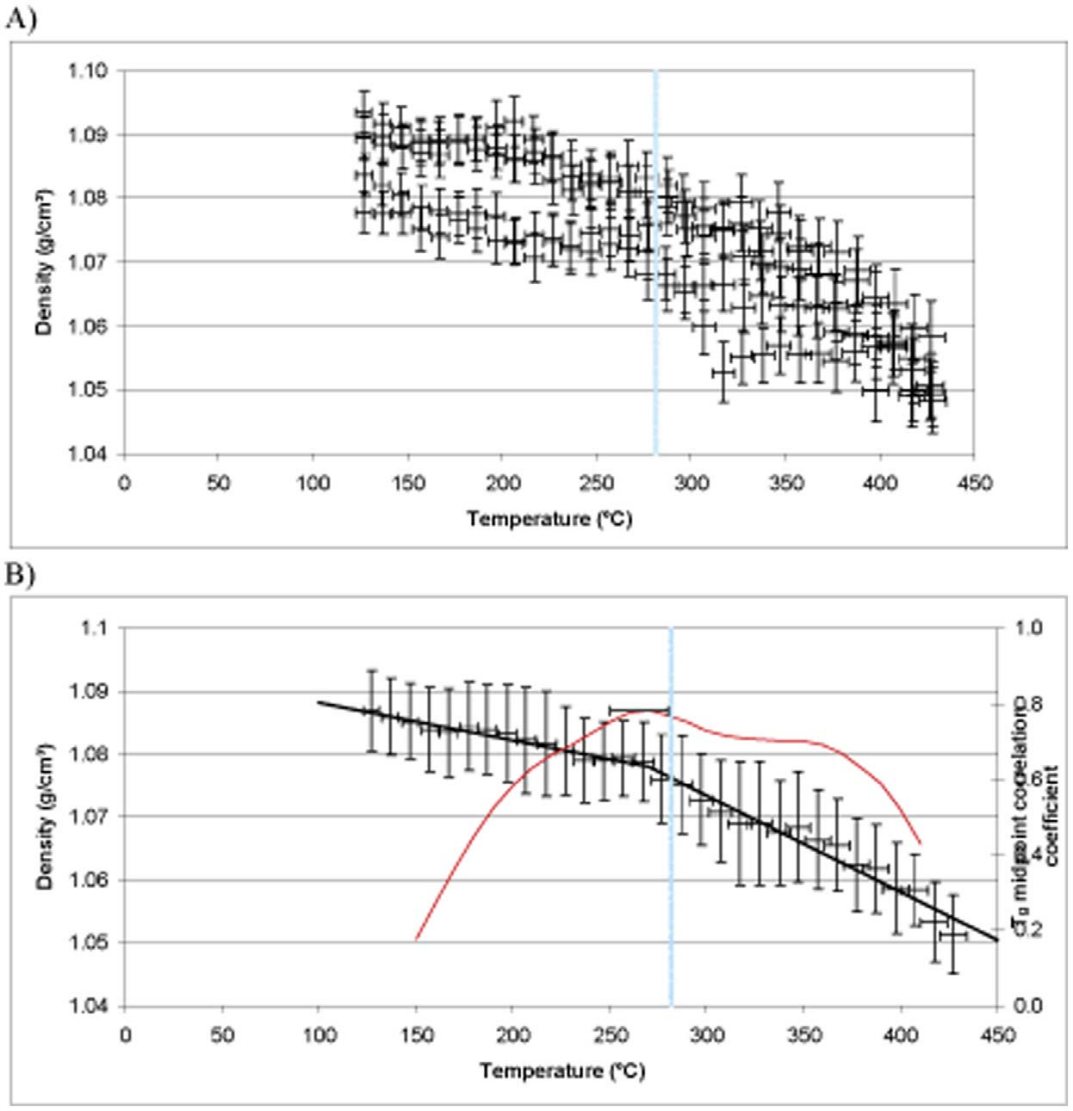
MD simulation for Epoxy 3 showing (A) raw data and (B) group data, experimental Tg superimposed in blue and prediction in red.

The DMTA results for Epoxies 1, 2 and 3 show T_g_ values of 208, 276 and 281°C respectively (Figure 10). The predictions for the same epoxy systems using MD simulation are displayed in Figures 11–13. The top 5% predicted peak for Epoxy 1 spans 200–225°C, with the best match at 215°C, in close agreement with the thermo-mechanical data and is unimodal in appearance giving confidence in the simulation. Epoxy 2 has a broad distribution spanning 220–280°C (top 5%), but with the best match at between 260°C. The peak maximum is in close agreement with the DMTA data. Simulations above 375°C appear to belong to a separate phenomena, perhaps thermal decomposition, and were excluded from analysis. Most interesting is Epoxy 3, which has a bimodal distribution with the top 5% of the major peak spanning 250–280°C, centred at 270°C. The shape of the simulated density vs. temperature plot clearly contains structural information relating to the nature of the epoxy system under study (*e.g.* crosslink density, free volume and rotational freedom, *etc.*).

### Conclusions

The methodology which has been developed for model design, construction and curing is now at a point where very high quality models are being produced. The process is now streamlined and mostly automated by using the Amorphous Cell module in Materials Studio and programs developed in-house. DMTA was performed on the three epoxy systems to determine the values of T_g_. The ultimate aim is to validate the models by using them to predict T_g_ within reasonable accuracy of the values measured empirically. Preliminary results involving the three epoxy systems are very encouraging with simulated values falling within 10–20 K of the experimental values. It should of course be borne in mind that as T_g_ is not a first order thermodynamic parameter there is also an uncertainly in the experimentally determined values, so the systematic error in both experimental and simulated values is not greater than 20K.
